# The Development of a Composite Thin Film Barrier of Tungsten Fe_3_O_4_-rGO (FerGO) for the Radiation Shielding of Medical Personnel

**DOI:** 10.3390/polym16020215

**Published:** 2024-01-12

**Authors:** Seon-Chil Kim, Jian Hou, Won-Gi Jang, Hong-Sik Byun

**Affiliations:** 1 Department of Medical Informatics, Keimyung University, 1095 Dalgubeol-daero, Daegu 42601, Republic of Korea; 2 Department of Biomedical Engineering, Keimyung University, 1095 Dalgubeol-daero, Daegu 42601, Republic of Korea; 3 School of Intelligent Manufacturing, Luoyang Institute of Science and Technology, Luoyang 471023, China; 4 Kwang Won Electronics, Yangsan-si 50590, Republic of Korea; 5 Department of Chemical Engineering, Keimyung University, 1095 Dalgubeol-daero, Daegu 42601, Republic of Korea; hsbyun@kmu.ac.kr

**Keywords:** radiation, graphene oxide, radiation exposure, X-ray, nanofiber

## Abstract

Tungsten is the most effective eco-friendly material used for radiation shielding in hospitals. However, despite its commendable density and shielding performance, tungsten faces challenges in miscibility with other materials because of its elevated melting point and strength. In this study, to protect medical personnel against scattered rays, which are indirect X-rays, a lightweight material was prepared by mixing graphite oxide material, considering its thinness and flexibility. Tungsten particles were evenly dispersed in the polymer, and nanofibers were prepared using this blended polymer solution via electrospinning. Concurrently, the process technology was explored to craft a thin film sheet and obtain a lead-like shielding effect. A spinning solution was prepared by mixing Fe_3_O_4_-rGO (FerGO) and tungsten. At 60 kVp, 0.1 mm was measured as 0.097 mmPb, at 80 kVp, 0.2 mm was measured as 0.196 mmPb, and at 100 kVp, 0.3 mm was measured as 0.279 mmPb, showing similar shielding performance to lead. As density directly affects the shielding effect, graphene oxide played an important role in increasing the density of the material from 1.941 g/cm^3^ to 2.302 g/cm^3^. Thus, this study provides an effective process for producing thin film sheets equivalent to lead.

## 1. Introduction

Recently, public interest in radiation exposure has increased due to the handling of the Fukushima nuclear power plant accident. Medical radiation used in medical institutions for diagnosis and treatment also affects the human body, but is used for the treatment of diseases. However, great care must be taken regarding the radiation exposure of the medical personnel using it [1]. Therefore, efforts have recently been made to develop shielding tools to reduce medical radiation exposure and develop eco-friendly shielding materials to reduce the risks of direct and indirect radiation exposure [2]. In particular, the radiation exposure of medical personnel is affected more by indirect radiation, or scattered radiation, generated in the space of medical practice than by direct radiation [3]. Scattered rays are sometimes excluded from the purpose of direct-ray shielding tools due to low-dose radiation, so the risk of radiation exposure is increasing [4].

Low-dose radiation, such as natural radiation, generally means less than 100 mSv [5]. Although it does not provide evidence of direct cancer occurrence at radiation exposure below 100 mSv, BEIR VII also supports the linear proportionality theory without a threshold, and cancer and genetic disorders are explained as being potential [6]. Therefore, even low-dose radiation requires an active defense from the user’s perspective.

There are limits to medical personnel’s protection against low-dose radiation. In particular, the area of the human body that does not interfere with medical procedures is limited. Parts such as the hands are required to be sensitive to sensations, so diagnosis and treatment are carried out with exposure of the hands and other areas to medical radiation during surgery. In addition, existing shielding tools are made of heavy metal hazardous materials such as lead, and, due to their weight, they are difficult to use in practice, so they are changing to thin films and eco-friendly materials [7,8]. The most used materials are tungsten, barium sulfate, and bismuth oxide, which are produced in the form of materials such as fibers, sheets, and plates [9].

Medical radiation shielding performance is directly related to the density and thickness of the shielding material and the atomic number of the material. Therefore, there are some limitations to improving shielding performance while satisfying the conditions of materials being thin film and lightweight, so research on types of shielding tools is necessary [10]. Therefore, in this study, rather than developing materials in the form of a general sheet, we aim to develop a thin-film sheet manufacturing technology that creates sheets in the form of nanofibers through electrospinning by mixing the shielding material used with graphene oxide material in a complex form. First, in this study, iron oxide and tungsten in the form of proposed graphene oxide were synthesized. This was because it is not easy to block radiation with the thin structure of a thin film, so we attempted to apply the characteristics of graphene, which has a multilayer–interlayer structure [11]. The density was 19.25 g/cm^3^ for tungsten and 5.25 g/cm^3^ for iron oxide. Both have excellent compatibility with polymer materials and durability. The materials are very stable against external influences such as sunlight, air moisture, and heat, making it easy to manufacture thin sheets [12,13]. Therefore, we attempted to produce a thin film sheet through electrospinning by mixing a composite with polyurethane. A thin barrier film of less than 0.1 mm could be processed into desired shapes such as hats, gloves, and aprons using fabric and applied to various shielding tools. Therefore, in this study, we comparatively evaluated the shielding performance based on standard lead and examined the feasibility of thin film sheets as a functional shielding tool that can guarantee the safety of medical personnel in medical institutions. 

## 2. Methods and Materials

### 2.1. Radiation Shielding Mechanism 

In medical institutions, low-dose radiation mainly refers to indirect radiation, that is, scattered radiation, rather than direct radiation [14]. However, for radiation shielding performance, the lead equivalent is used as the standard based on direct-line shielding [15]. Therefore, improvement in shielding performance refers to the amount of attenuation of the dose incident on the surface area. This can be explained by the incident photon radiation and its interaction with the shielding material. In general, shielding is defined as the attenuation of the intensity of incident radiation through interaction with the constituent atoms or nuclei of the medium when radiation passes through a material [16]. The degree of response is expressed as an exponential decay function, as shown in Equation (1). Therefore, it depends on the thickness and density within the space [17].

(1) $$A=A_{0}\exp(\mu x).$$

*A*_0_ represents the radiation intensity when there is no shielding material, μ is the linear attenuation coefficient (cm^−1^), and thickness (x) refers to the distance through which radiation passes per unit length through the shielding material [18].

(2) $$A=A_{0}\exp\left[-\left(\frac{\mu }{\rho }\right)\rho x\right].$$

The linear attenuation coefficient (μ) varies depending on the energy of the radiation and the type of shielding material, and, even for the same shielding material, it varies depending on the composition of the material. Therefore, shielding must be confirmed by introducing the mass attenuation coefficient ($\mu /\rho ,g/cm^{3}$) and density thickness ($\rho x,g/cm^{2}$), as expressed in Equation (2) [19]. However, in the case of scattering, the build-up factor (B) can be considered, and B varies depending on the thickness of the shielding material and the linear attenuation coefficient [20,21]. Therefore, the change in the intensity of radiation, considering the scattered rays, can be expressed by Equation (3). Therefore, the radiation shielding effect is affected by the density and thickness of the shielding material; if the thickness is identical, then the greater the density and the more positive the shielding effect from scattered rays [22].

(3) $$A=A_{0}B\left(\mu \chi \right)\exp\left[-\left(\frac{\mu }{\rho }\right)\rho x\right].$$

Considering these aspects, in this study, graphene oxide was used to reduce thickness and increase density during the shielding manufacturing process.

### 2.2. Materials 

Tungsten powder (Daegutech, Daegu, Republic of Korea, WP40) was purchased, washed with ethanol, and dried in an oven at 70 °C for 24 h, and sonication was performed for 1 h simultaneously with washing. For the synthesis of graphene oxide (GO), natural graphite, NaNO_3_ (≥99.0%), and KMnO_4_ (>99.0%) were purchased from Sigma-Aldrich (St. Louis, MO, USA); H_2_SO_4_ (95.0%) was purchased from Samchun, and H_2_O_2_ (35%) was purchased from J.T. Baker (Phillipsburg, NJ, USA). Each sample was used as received, without further purification. For the synthesis of Fe_3_O_4_-rGO (FerGO), FeCl_2_·4H_2_O (99.0%) was purchased from Samchun (Pyeongtaek-si, Republic of Korea), FeCl_3_·6H_2_O was purchased from Sigma-Aldrich, HCl (1N) was purchased from Duksan (Ansan-si, Republic of Korea), and NH_4_OH (29%) was purchased from J. T. Baker. To manufacture polyurethane (PU) nanofiber mats, the matrix polymer PU (K 20161) was purchased from Songwon Industries (Ulsan, Republic of Korea), and N, N-dimethylacetamide (DMAc, >99%) and MEK (>99.5%) were purchased from Duksan Pure Chemicals (Ansan-si, Republic of Korea). Each sample was used as received, without further purification. The treated tungsten powder was analyzed using X-ray fluorescence (Shimadzu, Kyoto, Japan, XRF-1800) for component analysis [23].

### 2.3. GO Synthesis

GO was prepared according to Hummer’s method, as follows [24]: Natural graphite powder, NaNO_3_, and H_2_SO_4_ were placed in a round-bottom flask and stirred. KMnO_4_ was slowly added while maintaining a temperature of 0 °C and stirred for 2 h. After the stirring process was complete, a 5 vol% sulfuric acid solution was added to the graphite residue to form a solution and, subsequently, H_2_O_2_ was added to obtain a yellow graphite dispersion. To remove unreacted residues, centrifugation was performed by adding sulfuric acid and hydrogen peroxide solutions, and washing was repeated several times with distilled water. After the washing process was completed, the graphite dispersion was completely dried at ambient temperature for more than 24 h in a vacuum oven to obtain graphite oxide as a brown powder, which is a bulk material of GO. The prepared graphite oxide powder (100 mg) was added to deionized (D.I.) water (100 mL) and completely dispersed by ultrasonic pulverization. Undispersed residues were removed by centrifugation, and sheet-shaped GO was obtained after drying in a vacuum oven [25].

### 2.4. Fe_3_O_4_-rGO(FerGO) Synthesis

**Figure 1:**
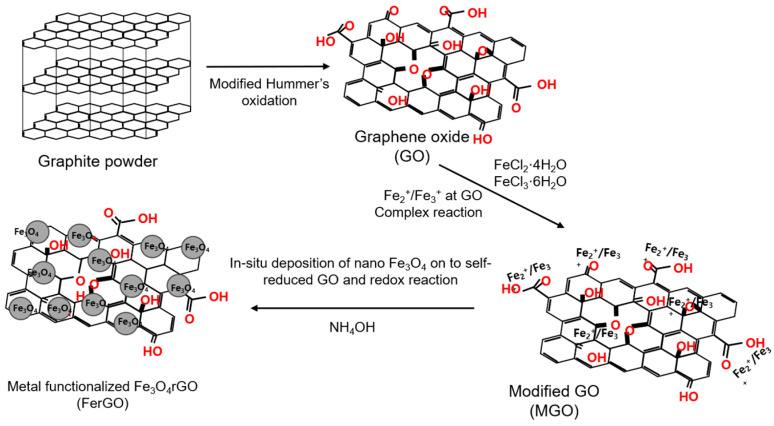
Schematic diagram of FerGO synthesis.

**Figure 2:**
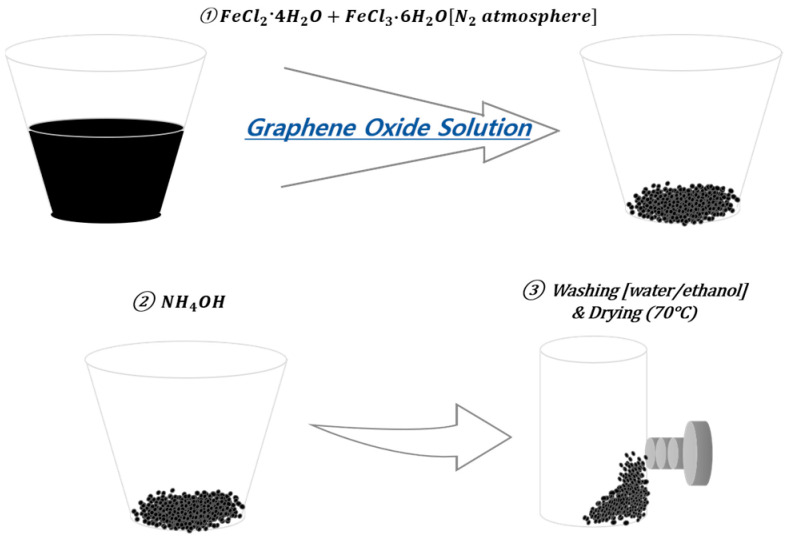
Pictures of FerGO synthesis.

GO sheets (100 mg) were placed in D.I. water (100 mL), completely dispersed via ultrasonic disintegration, and placed in a round flask. A mixed solution of FeCl_2_·4H_2_O (0.2 g) dissolved in HCl (0.5 mol, 5 mL) with FeCl_3_·6H_2_O (0.54 g) dissolved in DI water (10 mL) was slowly injected into the round flask under a nitrogen atmosphere and stirred for 15 min. After injecting 16 mL of NH_4_OH and stirring for 45 min, a reducing agent (hydrazine monohydrate) was added at 90 °C, and the reaction occurred for 2 h. After the reaction, the sheets were washed several times with ethanol and D.I. water and dried in a vacuum oven at 70 °C for more than 2 h to obtain Fe_3_O_4_-rGO in the form of a black powder [26,27]. Figure 1 presents a schematic diagram of the FerGO synthesis pathway and Figure 2 depicts photographs of the FerGO synthesis. Figure 2 visualizes the movement of FerGO according to the movement of the magnet, indicating that the Fe reacted well with the GO.

### 2.5. Manufacturing Thin Film Sheets Using Nanofibers

**Figure 3:**
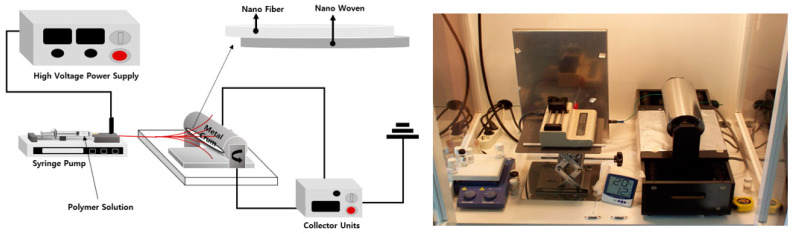
Schematic diagram of electrospinning and real equipment.

A thin-film sheet for medical radiation shielding was manufactured using nanofibers [28]. Nanofibers were manufactured by electrospinning after mixing the PU spinning solution with a shielding composite material in a ratio of 7:3. The composite material was manufactured using tungsten and FerGO in a ratio of 5:5. Figure 3 presents a schematic of the electrospinning process and a photograph of the electrospinning equipment.

First, the PU composite was mixed with a solution of DMAc and MEK at a ratio of 5:5. After completely dissolving the polymer by stirring for 24 h, it was placed in a 5 mL syringe and left upright for more than 30 min to completely remove any remaining air bubbles before use. The injection speed of the spinning solution (0.6 mL/h) was controlled using KDS100 (KD Scientific Inc. Holliston, MA, USA), and the spinning voltage (10 kV) was adjusted using PCS 60K02VIT (CHUNGPA EMT Co., Ltd., Bucheon-si, Republic of Korea). At this time, the needle size was 22 gauge and the distance was adjusted to less than 10 cm.

To confirm the synthesis of GO and FerGO, the surface structures were analyzed using Raman spectroscopy (WITec project 4.1, alpha 300R), FT-IR (Thermo Scientific, Waltham, MA, USA, IS50), and scanning electron microscopy (SEM, Hitachi, Tokyo, Japan, S-4800). The surface structures of GO and FerGO were analyzed using this method. Additionally, X-ray diffraction (XRD, Rigaku, Tokyo, Japan, D/Max-2500 V) was used to analyze the FerGO in the nanofibers [29]. To manufacture nanofibers, tungsten and FerGO were first mixed in a solvent of the matrix polymer PU, and, subsequently, sonication was used to prevent the agglomeration of the tungsten and induce a uniform dispersion of tungsten and FerGO. After about 5 min of sonication, PU was mixed while stirring at 600 rpm and, after 12 h, the final spinning solution was prepared. Nanofibers were manufactured using this spinning solution, and an eight-layer thin film was produced by hot pressing at 70 °C and 6000 PSI to obtain a thin film sheet. SEM was performed to compare the surface structure of nanofibers according to the composition of tungsten metal particles. The manufactured shielding material was observed using an optical microscope (FESEM; field emission scanning electron microscope, Hitachi, S-4800, JAPAN, 2011) [30].

### 2.6. Radiation Shielding Experiment

**Figure 4:**
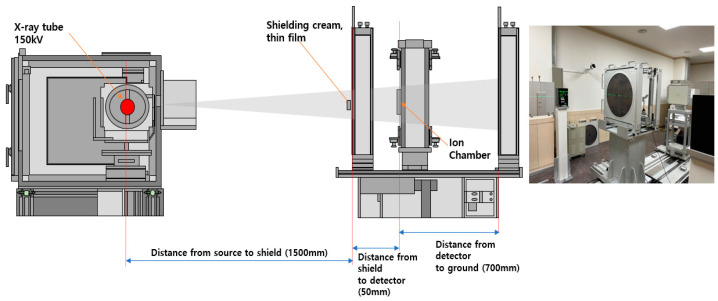
Schematic diagram of evaluation of shielding effect.

The shielding performance of the thin film sheets manufactured in this study was evaluated for medical radiation shielding. The shielding performance evaluation configuration was tested as illustrated in Figure 4.

The shielding performance of the manufactured thin film sheet was expressed in terms of the radiation shielding rate. The experiment was conducted by applying the lead equivalent test method for X-ray protection products (KS A 4025:2017), according to the regulations for X-ray protection products [31]. A radiation generator (X-ray tube, E7239X, Toshiba Electron tubes & Devices Co., Ltd., Tokyo, Japan) was used. The radiation dose detector was used after verification and calibration with a Radical Corporation Mo.9517 Radiation Monitor, Mo10×5-6, 6 cc Ion chamber (Radical Corp., Santiago de Surco, Peru). The effective tube voltage of the radiation generator was increased by 20 kVp from 60 kVp to 120 kVp, and irradiation was performed at 100 mA for 0.1 s (10 mAs). 

To increase the accuracy of measuring the shielding rate of the thin film sheet, the radiation dose was measured in the presence and absence of the thin film sheet. The shielding ratio was calculated using Equation (4), where *S* denotes the shielding rate of the thin film sheet, *D* is the radiation dose before penetration, and *D*_0_ is the radiation dose after penetration of the shielding film [32].

(4) $$S=\frac{D-D_{0}}{D}\times 100.$$

To calculate the mass attenuation coefficient ($\mu_{m}$), the line attenuation coefficient (μ) according to the thickness (x) of the shielding sheet was calculated using Equation (5). The mass attenuation coefficient was calculated by dividing the line attenuation coefficient by the density ($\rho_{c}$) using Equation (6) [33]. The line attenuation coefficient represents the radiation dose reduction by the interaction between the radiation and the sheet, depending on the thickness of the thin film sheet. The mass attenuation coefficient refers to the radiation dose reduction by the interaction between the shielding film and the radiation when the density and thickness are 1 [34].

(5) $$\mu =\frac{\left(\ln\frac{D_{0}}{D}\right)}{x},$$

(6) $$\mu_{m}=\frac{\mu }{\rho_{c}}.$$

## 3. Results

### 3.1. Synthesis Results of GO and FerGO

**Figure 5:**
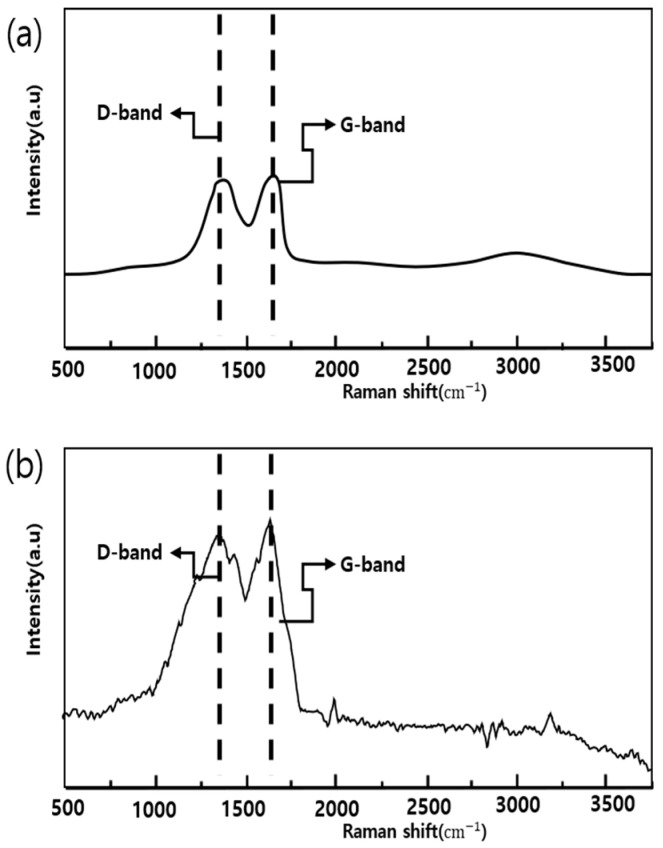
Raman analysis: (**a**) GO powder; (**b**) FerGO powder.

**Figure 6:**
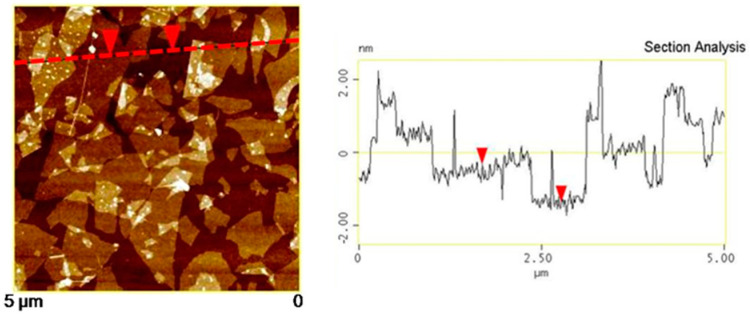
AFM image of GO and height profile (This is an image measuring the thickness. The dotted line is based on 0, and the two arrows indicate the thickness at the same point).

**Figure 7:**
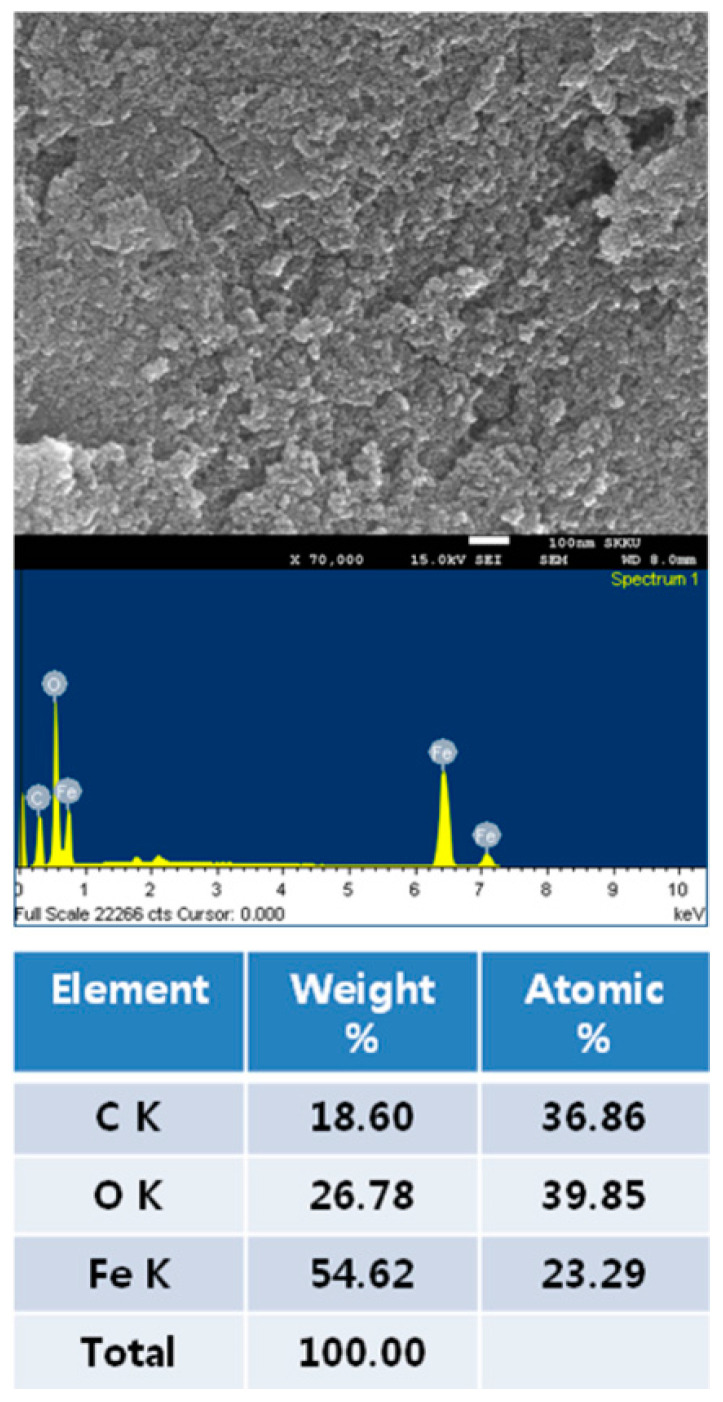
SEM (EDS) data of FerGO.

The results of the Raman analysis of the powder to confirm the synthesis of GO and FrGO are illustrated in Figure 5. The Raman analysis of GO revealed a D-band observed at 1.353 cm^−1^ and a G-band observed at 1594 cm^−1^. For the FerGO powder, the D-band and G-band were observed at 1357 cm^−1^ and 1598 cm^−1^, similar to those of the GO powder. Moreover, AFM analysis, the results of which are presented in Figure 6, was performed to validate the GO thickness. Consequently, the partially overlapping and folded parts of the GO were simultaneously confirmed, and the thickness of the GO in a single layer was approximately 1 nm. Additionally, SEM and EDS analyses, the results of which are presented in Figure 7, confirmed the presence of Fe_3_O_4_ in FerGO, and the atomic percentages of C, O, and Fe were 36.86, 39.85, and 23.29%, respectively.

**Figure 8:**
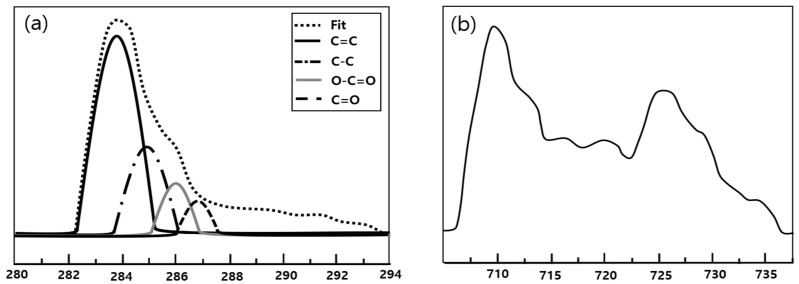
XPS data of FerGO: (**a**) C1s; (**b**) Fe2p.

The results of the XPS analysis of the surface bonding of GO and FerGO are presented in Figure 8. As shown in the figure, the single bond (286.6 eV) and double bond (288 eV) of carbon and oxygen were significantly reduced owing to the removal of the oxygen group surrounding the graphene after reduction by either hydrazine or by the self-reduction of GO. Notably, a new single bond (285.4 eV) between carbon and nitrogen was created by adding hydrazine (N_2_H_4_), a reducing agent. In particular, the XPS results indicated that the metal oxide was well bonded to the GO. In Figure 8, the Fe_2_P XPS spectra of FerGO reveal peaks at 711.3, 711, and 711.2 eV of Fe_2_P_3/2_ and peaks at 724.5, 724.2, and 724.4 eV of Fe_2_P_1/2_. These binding energy results are consistent with the general Fe_3_O_4_ XPS results.

**Figure 9:**
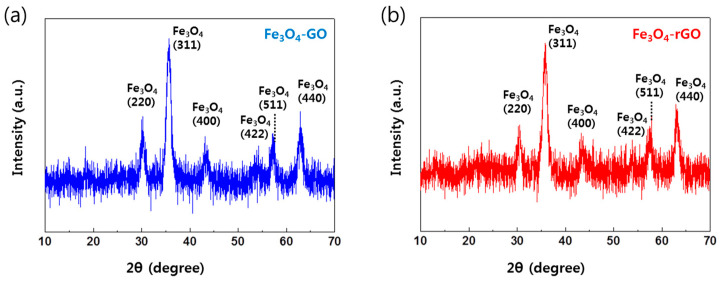
X-ray diffraction (XRD) results of (**a**) FeGO and (**b**) FerGO.

Moreover, XRD was performed to confirm the reaction of Fe_3_O_4_ with GO to produce FerGO. Figure 9 shows the results of the XRD analysis. As shown in Figure 9a, the surface of the Fe_3_O_4_ peaks, i.e., (220), (311), (400), (422), (511), and (440), were observed at 30.24°, 35.72°, 43.17°, 53.53°, 57.18°, and 62.90°, respectively, which is similar to those reported before for Fe_3_O_4_ nanoparticles [35,36]. These results were also observed in the XRD of FerGO, indicating that Fe was not lost in FerGO. The XRD result of the FerGO can thus be concluded to confirm the success of the FerGO synthesis.

**Figure 10:**
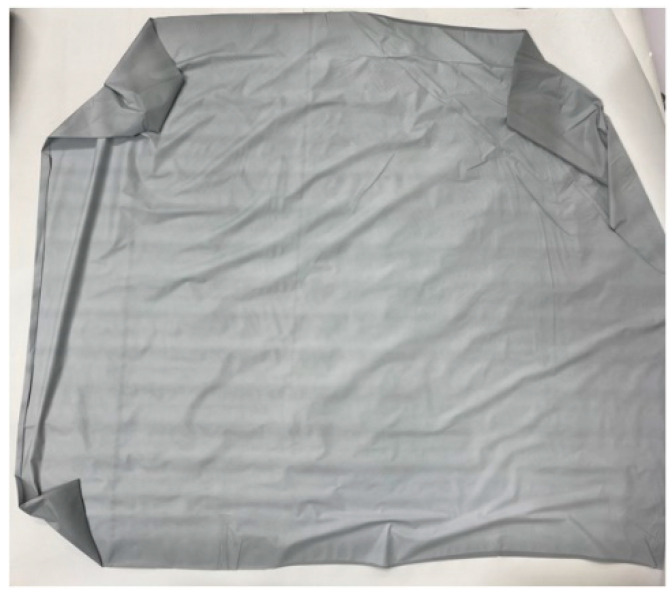
Appearance of the manufactured thin film sheet.

**Figure 11:**
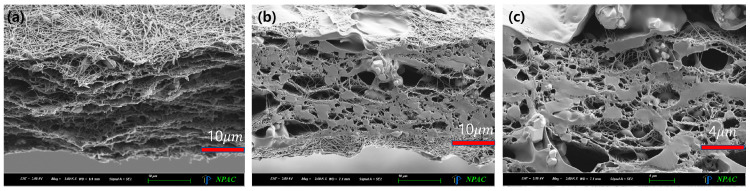
Enlarged cross-sectional image of (**a**) the external appearance of a 0.3 mm thick sheet, (**b**) the distribution of metal particles, and (**c**) the surface and cross-section by enlarging one part.

The physical characteristics and appearance of the thin film sheets produced for medical radiation shielding are listed in Table 1 and Figure 10. They are characterized as thin sheets that ensure lightweight conditions and allow for user activities. In addition, the density of Type B with added GO iron oxide of graphene oxide is higher. As illustrated in Figure 11, the distribution of metal particles within the thin film sheet is enlarged for visible inspection, revealing a well-distributed dispersion of iron oxide in the tungsten particles and graphene oxide.

### 3.2. Evaluation of the Shielding Performance of Thin Film Sheets and Standard Lead

The performance of the manufactured thin film sheets was evaluated and compared, as shown in Table 2 and Table 3. A comparative evaluation was conducted with standard lead, and the shielding performance is presented based on the lead equivalent. In the case of the presented 0.1 mm thin film sheet, the same performance was observed at 100 kV, exhibiting the same shielding performance as lead in the low-energy range. Overall, the evaluated shielding performance was expressed consistently and well, without significant changes, indicating that the process technology is effective for particle dispersion and uniformity during the manufacturing process.

## 4. Discussion

The maximum tube voltage energy for radiation shielding in medical institutions is limited to 120 kVp, which facilitates the manufacture of customized energy-shielding materials [37,38]. In general, shielding materials are manufactured based on lead but are expressed in terms of lead thickness. This is known as the lead equivalent [39]. Therefore, shielding suits used in medical institutions are typically designed based on a lead equivalent of 0.25 mmPb. While existing shielding suits, crafted from flexible sheets combining lead and rubber powder, offer satisfactory wear properties, their weight poses a hindrance to routine activities [40]. Therefore, there is a growing demand for lightweight alternatives.

Eco-friendly shielding materials are emerging to overcome the weight and toxicity of lead; however, they have various limitations such as economic efficiency, complexity of the manufacturing technology, and affinity with polymer materials [41]. Among these challenges, the most difficult aspect is the need for mass production technology for shielding clothing sheets that can shield with a thickness equivalent to lead, ensuring the flexibility and activity of the product.

In this study, we attempted to produce a thin film sheet for medical radiation shielding using graphene oxide structure and nanofibers to provide a shielding performance equivalent to that of Pb. However, owing to the limitations of the processing method and the electrospinning of tungsten, a representative eco-friendly material, considerable research is needed to achieve an economic effect through mass production. To reduce the thickness, the density must be increased, and to increase the density, the porosity between the particles must be lowered. Therefore, the dispersion technology of the shielding material determines the density structure [42]. In this study, FerGO mixed in the spinning solution contributed greatly to the improvement in shielding performance based on density improvement. This study anticipates that the shielding performance of future shields can be reduced by increasing the density and flexibility of the developed material to ensure user activity. To satisfy the thin film sheet production conditions suggested by the results of this study, the material properties of FerGO were analyzed. Therefore, we believe that no problems will be encountered in planning the mass production process. In addition, it has excellent miscibility with tungsten materials; therefore, we expect that no problems will be encountered in the production of shielding films. Therefore, manufacturing the spinning solution necessary for manufacturing the thin film shield is expected to be easy and the physical properties of the manufactured sheet are expected to be excellent.

A limitation of the research process in this study is that the synthesis of the shielding material was applied only to graphene oxide and iron oxide. It failed to account for the need for various syntheses tailored to specific causes and conditions, utilizing tungsten and iron oxide as primary materials. In medical settings, radiation exposure can occur unexpectedly in areas where radiation is generated, necessitating permanent shielding clothing for everyday life. This study addresses this need by developing a thin film sheet with shielding performance equivalent to lead, contributing to a safer medical environment. 

## 5. Conclusions

A lightweight thin film sheet was manufactured as a shield against low-dose radiation in medical institutions. Electrospinning was performed using a solution of Fe_3_O_4_-rGO (FerGO) and tungsten, yielding nanofibers. The measurements at 60 kVp, 80 kVp, and 100 kVp indicated lead equivalent thicknesses of 0.097 mmPb, 0.196 mmPb, and 0.279 mmPb, respectively, demonstrating a similar shielding performance to lead. Furthermore, the introduction of graphene oxide using Fe_3_O_4_-rGO (FerGO) resulted in an increased density of 2.302 g/cm^3^, compared to the general tungsten thin film sheet with a density of 1.941 g/cm^3^. Consequently, the shielding rate of the electrospun thin film sheet with graphene oxide exhibited significant improvement.

## Figures and Tables

**Table 1:** Physical characteristics of 0.3 mm thin film sheets.

Type *	Weight (kg/m^2^)	Shielding Material Weight (kg/m^2^)	Thickness (mm)	Density (g/cm^3^)
A	0.525 ± 0.020	0.183 ± 0.028 (Tungsten only)	0.300 ± 0.020	1.941 ± 0.065
B	0.652 ± 0.034	0.104 ± 0.028 (Tungsten and FerGO)		2.302 ± 0.142

* Type A: General nanofiber; Type B: Graphene oxide nanofibers.

**Table 2:** Evaluation of shielding performance of standard lead (0.1 mm, 0.2 mm, 0.3 mm).

mmPb	Transmission Dose	60 kVp		80 kVp		100 kVp		120 kVp	
		None	Lead	None	Lead	None	Lead	None	Lead
0.1	Dose (μR)	420.15	74.74	886.78	261.25	1521.23	567.27	1997.75	827.87
	Shielding rate (%)	82.21		70.54		62.71		58.56	
0.2	Dose (μR)	420.15	22.86	886.78	125.92	1521.23	326.46	1997.75	475.26
	Shielding rate (%)	94.56		85.89		78.54		76.21	
0.3	Dose (μR)	420.15	8.49	886.78	70.85	1521.23	204.76	1997.75	293.26
	Shielding rate (%)	97.98		92.01		86.54		85.32	

**Table 3:** Evaluation of shielding performance of thin film sheets (0.1 mm, 0.2 mm, 0.3 mm).

Thickness (mm)	Transmission Dose	60 kVp		80 kVp		100 kVp		120 kVp	
		Non	SB	Non	SB	Non	SB	Non	SB
0.1	Dose (μR)	420.15	83.53	886.78	243.51	1521.23	589.63	1997.75	874.42
	Shielding rate (%)	80.12		72.54		61.24		56.23	
	Lead equivalent (mmPb)	0.097		0.103		0.098		0.096	
0.2	Dose (μR)	420.15	34.23	886.78	110.76	1521.23	228.80	1997.75	324.04
	Shielding rate (%)	91.85		87.51		84.96		83.78	
	Lead equivalent (mmPb)	0.206		0.196		0.185		0.182	
0.3	Dose (μR)	420.15	19.24	886.78	51.34	1521.23	106.79	1997.75	188.19
	Shielding rate (%)	95.42		94.21		92.98		90.58	
	Lead equivalent (mmPb)	0.305		0.293		0.279		0.283	

## Data Availability

Data are contained within the article.
